# Traditional Chinese medicine in thyroid-associated orbitopathy

**DOI:** 10.1007/s40618-023-02024-4

**Published:** 2023-02-12

**Authors:** Y. P. Hai, A. C. H. Lee, K. Chen, G. J. Kahaly

**Affiliations:** 1 Molecular Thyroid Research Laboratory, Department of Medicine I, Johannes Gutenberg University (JGU) Medical Center, Langenbeckstreet 1, 55131 Mainz, Germany; 2 Department of Endocrinology, Hainan General Hospital, Hainan Affiliated Hospital of Hainan Medical University, Haikou, China; 3 Division of Endocrinology and Metabolism, Department of Medicine, LKS Faculty of Medicine, The University of Hong Kong, Queen Mary Hospital, Hong Kong SAR, China

**Keywords:** Traditional Chinese medicine, thyroid-associated orbitopathy, Orbital fibroblasts, Autophagy, Inflammation, Oxidative stress

## Abstract

**Purpose:**

Orbital fibroblasts (OF) are considered the central target cells in the pathogenesis of thyroid-associated orbitopathy (TAO), which comprises orbital inflammation, orbital tissue edema, adipogenesis, fibrosis, oxidative stress and autophagy. Certain active ingredients of traditional Chinese medicine (TCM) demonstrated inhibition of TAO-OF in pre-clinical studies and they could be translated into novel therapeutic strategies.

**Methods:**

The pertinent and current literature of pre-clinical studies on TAO investigating the effects of active ingredients of TCM was reviewed using the NCBI PubMed database.

**Results:**

Eleven TCM compounds demonstrated inhibition of TAO-OF in-vitro and three of them (polydatin, curcumin, and gypenosides) resulted in improvement in TAO mouse models. Tanshinone IIA reduced inflammation, oxidative stress and adipogenesis. Both resveratrol and its precursor polydatin displayed anti-oxidative and anti-adipogenic properties. Celastrol inhibited inflammation and triptolide prevented TAO-OF activation, while icariin inhibited autophagy and adipogenesis. Astragaloside IV reduced inflammation via suppressing autophagy and inhibited fat accumulation as well as collagen deposition. Curcumin displayed multiple actions, including anti-inflammatory, anti-oxidative, anti-adipogenic, anti-fibrotic and anti-angiogenic effects via multiple signaling pathways. Gypenosides reduced inflammation, oxidative stress, tissue fibrosis, as well as oxidative stress mediated autophagy and apoptosis. Dihydroartemisinin inhibited OF proliferation, inflammation, hyaluronan (HA) production, and fibrosis. Berberine attenuated inflammation, HA production, adipogenesis, and fibrosis.

**Conclusions:**

Clinical trials of different phases with adequate power and sound methodology will be warranted to evaluate the appropriate dosage, safety and efficacy of these compounds in the management of TAO.

## Introduction

Thyroid-associated orbitopathy (TAO), also termed Graves’ orbitopathy, is an orbital inflammatory disorder related to autoimmune thyroid disease and is the most common extrathyroidal manifestation of Graves’ disease (GD) [1–5]. The classical features of TAO include soft tissue inflammation, upper eyelid retraction, diplopia, proptosis, as well as rare but sight-threatening complications due to dysthyroid optic neuropathy or corneal breakdown [6, 7]. Owing to significant disfigurement and disability, TAO compromises patients’ psychological well-being and incurs a huge socioeconomic burden [8–12]. Over the past two decades, important pharmacological advances in the management of TAO remarkably improved patients’ outcomes and quality of life. However, the current treatment options have limitations in terms of response rates, potential toxicities, geographical availability, and affordability, therefore the search for novel treatment strategies is warranted and ongoing [5, 13–17]. Importantly, various active ingredients of traditional Chinese medicine (TCM) demonstrated potential therapeutic benefits in in-vitro and animal studies of TAO. In this review, we provide a brief overview of the pathogenesis of TAO and summarize how these TCM compounds may provide novel therapeutics insights in TAO.

## Pathophysiology of TAO

The exact pathogenesis of TAO remains unclear, although most researchers have considered TAO an orbital inflammatory disorder related to autoimmune thyroid disease [15, 18–20]. The pathological hallmarks of TAO include orbital inflammatory infiltration, over-production of hydrophilic glycosaminoglycans, de novo adipogenesis, and tissue fibrosis. In the presence of genetic, autoimmune and environmental factors, autoimmune T cells, B cells, and OF are activated by unbalanced immune tolerance, resulting in a series of inflammatory responses, including cellular and humoral immunity [21, 22]. The overproduction of hydrophilic glycosaminoglycans, especially hyaluronan (HA), leads to orbital tissue edema, which, together with de novo adipogenesis, causes orbital tissue expansion [6, 18, 23, 24]. These pathological processes explain most of the clinical manifestations of TAO.

Orbital fibroblasts (OF) are considered the central cells in the pathogenesis of TAO [6, 15, 18, 19, 23, 24]. OF-mediated interaction with immune cells via the production of different cytokines and chemokines is the primary mechanism for maintaining orbital inflammation in TAO [24, 25], and most pathological processes and clinical features of TAO involve OF. Thyrotropin receptor (TSH-R) acts as the principal autoantigen in GD and TAO. TSH-R autoantibody (TSH-R-Ab) is the specific biomarker and major pathogenic autoantibody in these disorders, and its titer was positively correlated with the activity and severity of TAO [26–32]. Upon stimulation by TSH-R-Ab, TSH-R/insulin growth factor-1 receptor (IGF-1R) crosstalk signaling pathway represents the most important mechanism of OF activation, including overproduction of HA [18, 19], differentiation into adipocytes [23, 24], and differentiation into myofibroblasts induced by transforming growth factor (TGF)-β, interleukin (IL)-17A and IL-23A resulting in tissue remodeling and fibrosis [18, 19]. In addition, the activation of OF also produces cytokines and chemokines (e.g. IL-6, IL-8, IL-16, and monocyte chemoattractant protein-1 [MCP-1]), and perpetuates orbital inflammation [33].

Oxidative stress, defined as an imbalance between the production and elimination of reactive oxygen species (ROS), plays a major role in TAO [18, 34–36]. Oxygen radicals induced the proliferation of OF and the expression of 72-kDa heat shock protein, leading to the production of ROS and oxidative stress [18, 34, 35]. In contrast to healthy controls, various substances involved in oxidative stress, including superoxide dismutase, superoxide anions, malondialdehyde, hydrogen peroxide, and glutathione reductase, were significantly increased in the OF of patients with TAO (TAO-OF) [34]. Cigarette smoking, which is the most important environmental risk factor of TAO, enhanced the in-vitro ROS synthesis and suppressed the antioxidant machinery [7, 18]. Selenium, an antioxidant, reduced the proliferation of OF and the production of glycosaminoglycans and hyaluronan, and benefited patients with mild TAO [37, 38]. In addition, several other antioxidants, including beta-carotene, N‑acetyl cysteine, vitamin C, and melatonin, demonstrated therapeutic efficacy in in-vitro studies of TAO-OF [37, 39].

Autophagy, a natural and destructive mechanism that allows orderly degradation and recycling of unnecessary cellular components, has also been implicated in the pathogenesis of TAO recently [40]. Autophagy plays an adaptive role in cell survival, development, differentiation and intracellular homeostasis [41]. Autophagy is recognized as a ‘self-cannibalizing’ process that is active during stress (e.g. starvation, chemotherapy, infection, aging, and hypoxia) to protect organisms from various irritants and to regenerate materials and energy. However, autophagy can also lead to a form of programmed cell death distinct from apoptosis [41]. In the immune system, autophagy regulates antigen uptake and presentation, pathogen removal, immune cell survival, and cytokine-dependent inflammation [42]. Insufficient and excessive autophagic activities have been implicated in several autoimmune diseases, such as rheumatoid arthritis, systemic lupus erythematosus (SLE), Crohn’s disease, and multiple sclerosis [43]. A study showed that autophagy, induced by the pro-inflammatory cytokine IL-1β, was upregulated in TAO orbital tissues and blocking autophagy inhibited adipogenic differentiation [40]. Therefore, inhibiting autophagy may become a therapeutic target in TAO.

In summary, OF are mainly involved in orbital inflammation, orbital tissue expansion, adipogenesis, fibrosis, oxidative stress and autophagy in TAO. Inhibiting TAO-OF by interrupting one or more of the above pathological processes represents a major direction in developing novel therapeutic strategies.

## Potential therapeutic effects of TCM active ingredients in TAO

**Fig. 1:**
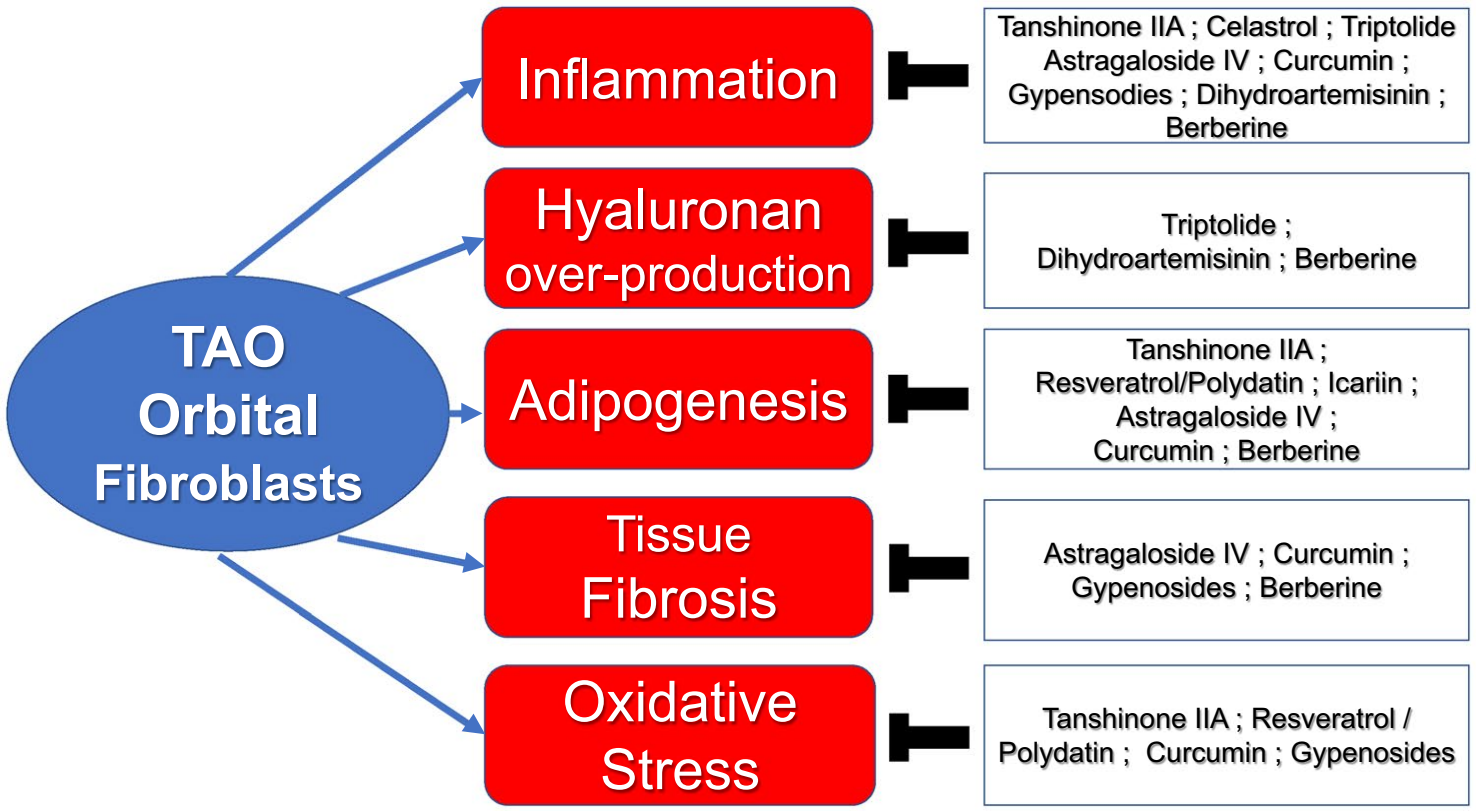
Active ingredients in traditional Chinese medicine: mechanisms of action shown in pre-clinical TAO studies

Eleven active ingredients of traditional Chinese medicine demonstrated potential therapeutic benefits in in-vitro experiments using OF, and three of them (polydatin, curcumin, and gypenosides) showed favorable effects in TAO mouse models. They are reviewed in this session and the mechanisms of action of these compounds in relation to the pathophysiology of TAO is summarized in Fig. 1.

### Tanshinone IIA

Tanshinone IIA, a lipophilic diterpene, is the most abundant active ingredient extracted from the Chinese herb *Salvia miltiorrhiza bunge* (Danshen) and it has been widely used to treat ischemic heart disease and cerebrovascular disease in the Chinese community for many centuries, owing to its proposed anti-inflammatory and anti-oxidative properties [44].

Tanshinone IIA demonstrated anti-inflammatory, anti-oxidative and anti-adipogenic effects on TAO-OF in an in-vitro study [45]. Tanshinone IIA inhibited IL-1β-induced expression of pro-inflammatory cytokines (IL-6, IL-8, and MCP-1), reduced cigarette smoke extract or H2O2-induced generation of ROS, and upregulated the expression of the anti-oxidative enzyme heme oxygenase-1 (HO-1) through activating extracellular signal-regulated kinase (ERK) signaling pathway. The expression of adipogenesis-related factors (peroxisome proliferator-activated receptor gamma, PPARγ; CCAAT-enhancer-binding protein alpha, C/EBPα) was downregulated. These results were consistent with findings from recent non-OF in-vitro studies, which confirmed the anti-inflammatory, anti-oxidative, as well as antifibrotic properties of tanshinone IIA [44, 46, 47].

### Resveratrol

Resveratrol, a well-known polyphenol phytoalexin, is extracted from the roots of the Chinese herb *Polygonum cuspidatum* (Huzhang) and multitudinous fruits (e.g. grapes, and berries). It has been extensively utilized and prescribed in TCM practice [48, 49]. Resveratrol demonstrated immunomodulatory effects in-vitro via various signaling pathways (e.g. arachidonic acid, nuclear factor kappa B [NF-κb], mitogen-activated protein kinase (MAPK) and anti-oxidant defense pathways [50]). Multiple studies employing animal models of inflammatory bowel disease showed that resveratrol exerted anti-inflammatory action via targeting various molecular targets and signaling pathways (e.g. NF-κb, Sirtuin-1 [SIRT1], mammalian target of rapamycin [mTOR], hypoxia-inducible factor-1α [HIF-1α], microRNA [miRNAs], nuclear factor erythroid 2-related factor 2 [NRF2], tumor necrosis factor-alpha [TNFα], and autophagy) [51]. In two double-blind placebo-controlled randomized clinical trials, supplementation with resveratrol improved clinical disease activity and quality of life in patients with ulcerative colitis, via reducing inflammation and oxidative stress [52, 53].

Treatment with resveratrol attenuated oxidative stress and suppressed adipogenesis in TAO-OF [54]. Resveratrol inhibited ROS production stimulated by cigarette smoke extract and H2O2. It reduced levels of HO-1, copper/zinc-superoxide dismutase [SOD], catalase and thioredoxin, but increased the level of manganese-SOD, reflecting regulation in the expression of anti-oxidative defenses. Resveratrol counteracted rosiglitazone (PPARγ agonist) induced adipogenesis, as evidenced by a decreased number of adipocytes and the accumulation of intracellular lipid droplets. Furthermore, alterations in the levels of transcriptional regulators suggested that resveratrol modulated ERK, JNK and NF-κB signaling pathways.

### Polydatin

Polydatin, a glycoside and natural precursor of resveratrol, similarly displayed anti-oxidative and anti-adipogenic properties in both in-vitro and in-vivo models [55]. It inhibited H2O2-induced ROS production by cultured non-TAO-OFs in a dose-dependent manner, while silencing NRF2 decreased the anti-oxidative effect of polydatin. In a TAO mouse model, treatment with polydatin reduced ROS production. The attenuation in orbital oxidative stress was associated with a lower level of Keap1, as well as a higher level of nuclear located NRF2 and antioxidant genes (e.g. NAD(P)H dehydrogenase, quinone 1 [NQO1]), suggesting that polydatin mediated anti-oxidative effect via stimulating Keap1/NRF2/ARE pathway. Polydatin also inhibited adipose tissue expansion and lipid droplet accumulation in retrobulbar fat of TAO mice, reflecting suppression of adipogenesis.

### Celastrol

Celastrol, a natural triterpene, is isolated from the root extracts of *Tripterygium wilfordii* and *Tripterygium regelii*, both from the Celastraceae family. It suppressed inflammatory responses in experimental models of various autoimmune diseases, such as rheumatoid arthritis, multiple sclerosis, inflammatory bowel disease, SLE, psoriasis and type 1 diabetes [56]. The potential mechanisms of celastrol-mediated anti-inflammatory and anti-oxidative effects include preventing the production and expression of pro-inflammatory cytokines, promoting the heat-shock response, inhibiting the generation of inducible nitric oxide synthase and lipid peroxidation, as well as restoring the level of autophagy [57].

In an in-vitro study [58], celastrol significantly reduced IL-1β-induced expression of pro-inflammatory molecules (IL‑6, IL‑8, prostaglandin E2, cyclooxygenase-2 [COX-2], intercellular adhesion molecule‑1 [ICAM-1]) in TAO-OF. The anti-inflammatory action of celastrol was mediated through inhibition of the NF-κB signaling pathway, as evidenced by suppression of IL-1β induced phosphorylation of IκBα (an inhibitor of NF-κB). This is consistent with the observation that celastrol attenuated inflammation in experimental models of osteoarthritis and rheumatoid arthritis by inhibiting the NF-κB signaling pathway [57, 59].

### Triptolide

Triptolide, a diterpene triepoxide, is another pharmacologically active component of *Tripterygium wilfordii* Hook F from the Celastraceae family. It has been used as a remedy for various inflammatory and autoimmune disorders, including lupus nephritis, rheumatoid arthritis, inflammatory bowel disease, and asthma [60]. In an in-vitro study, triptolide effectively inhibited interferon-γ induced activation of TAO-OF, as evidenced by reduced expression of human leukocyte antigen (HLA)-DR, ICAM-1, and CD40, as well as suppression of cellular proliferation and HA synthesis [61].

### Icariin

Icariin, a flavonoid isolated from several species of plants in the genus *Epimedium*, has several pharmacological properties, such as immunomodulatory, anti-inflammatory, anti-oxidative and lipid-lowering effects [62, 63]. Adipogenesis and autophagy were considered two important mechanisms in TAO [18, 24, 40]. Adipogenesis involves the process of autophagy, which is regulated by two factors, mTOR and 5' adenosine monophosphate-activated protein kinase (AMPK, a serine-threonine kinase that functions as a metabolic sensor and activates autophagy in response to low energy levels) [64]. Icariin attenuated cardiomyocyte hypertrophy induced by isoproterenol in mice, by suppressing apoptosis and promoting autophagic flux. Isoproterenol promoted phosphorylation of AMPK (p-AMPK) and inhibited phosphorylation of mTOR (p-mTOR), while icariin reversed these effects, suggesting that the cardioprotective effect was mediated through AMPK/mTOR signaling pathway [62].

The effects of Icariin were evaluated in both in-vitro study and in-vivo TAO mouse model [64]. Icariin inhibited the differentiation of preadipocytes into adipocytes through the suppression of autophagy, which was a key process essential for adipogenesis. Similarly, it suppressed adipogenesis in orbital tissues in terms of PPARγ expression and lipid droplet accumulation, while it partially reversed the enhanced autophagy observed in TAO mice. The inhibition of adipogenesis by icariin, which decreased p-AMPK and increased p-mTOR levels in both in-vitro and in-vivo studies, was probably mediated by the suppression of autophagy through AMPK/mTOR signaling pathway. Furthermore, Pingmu decoction (composed of a mixture of herbs including *Epimedium Brevicornu Maxim*) reduced viability/proliferation of TAO-preadipocytes and induced apoptosis of TAO-adipocytes, by activating programmed cell death via Fas/Fas ligand signaling pathway [65, 66].

### Astragaloside IV

The Chinese herbs *Radix Astragali Mongolici* (Huangqi), the dried root of leguminous plants Mongolia, is widely prescribed to treat cardiovascular disorders, hepatitis, kidney disease, and skin diseases in China [67, 68]. Astragaloside IV, a tetracyclic triterpenoid saponin, has been identified as one of the bioactive ingredients in Huangqi [67]. It has been reported to possess anti-oxidative, cardioprotective, anti-inflammatory, antimicrobial, antifibrotic, anti-diabetic, and immunoregulatory properties [69]. Astragaloside IV was effective in inhibiting pro-inflammatory macrophages and promoting the pro-resolving macrophages to ameliorate experimental inflammatory bowel disease via the regulation of the STAT signaling pathway [69]. Astragaloside IV improved oxidative stress-mediated endothelial dysfunction relevant to cardiovascular diseases through several mechanisms: preventing the uncoupling of endothelial nitric oxide synthase (eNOS), increasing eNOS and nitric oxide (NO), and enhancing several activating enzymes to activate the antioxidant system [70].

Astragaloside IV demonstrated an anti-inflammatory effect, which was mediated by suppression of autophagy, in both in-vitro and in vivo models of TAO [71]. It inhibited IL-1β-induced expression of pro-inflammatory cytokines (IL-6, IL-8, TNF-α, and MCP-1) in non-TAO-OF. Rapamycin (an autophagy activator) significantly enhanced, but autophagy inhibitors or silencing autophagy-related proteins attenuated inflammation of non-TAO-OF, suggesting that autophagy was involved in IL-1β mediated inflammatory response. Pre-treatment with astragaloside IV strongly inhibited IL-1β induced autophagy and prevented rapamycin from enhancing IL-1β mediated inflammation. In the TAO mouse model, astragaloside IV administration resulted in lower serum levels of thyroid hormones, TSH-R-Ab and pro-inflammatory cytokines. The orbital tissues of astragaloside IV-treated TAO mice also displayed significant improvement, as evidenced by a reduction in fat accumulation, collagen deposition, macrophage infiltration, as well as autophagic activity.

### Curcumin

Curcumin is an active ingredient extracted from *Curcuma longa*, a traditional Chinese medicinal herb with a long history of use as a treatment for inflammatory conditions in China and Southeast Asia [72]. Curcumin has strong anti-oxidative and anti-inflammatory activities and was tested in more than 100 clinical trials in various chronic diseases, including inflammatory bowel disease, rheumatoid arthritis and psoriasis [72].

The effects of curcumin on TAO-OF were evaluated in two separate in-vitro studies. Curcumin inhibited the production of proinflammatory cytokines induced by IL-1β (IL-6, IL-8, MCP-1, ICAM-1). Upon induction of adipogenic differentiation, curcumin significantly reduced intracellular lipid droplet accumulation and levels of adipogenic transcription factors (PPARγ, C/EBPα, and C/EBPβ), reflecting suppression of adipogenesis. H2O2 or cigarette smoke extract stimulated ROS production was also attenuated by pretreatment with curcumin. Curcumin inhibited phosphorylation of multiple signaling molecules (ERK, JNK, NF-κB, p65), and stimulated nuclear translocation of β-catenin during adipogenesis which probably resulted in increased expression of downstream anti-adipogenic genes [73].

In another study curcumin demonstrated anti-fibrotic and anti-angiogenic properties [74], Transforming growth factor beta 1 (TGFβ1) induced expression of myofibroblast differentiation markers (connective tissue growth factor, CTGF; alpha-smooth muscle actin, α-SMA) and phosphorylation of SMAD2/3 (major signal transducers for receptors of TGFβ superfamily) were suppressed by curcumin, therefore curcumin inhibited TGFβ1 induced myofibroblast differentiation of TAO-OF. The conditioned medium from curcumin-treated TAO-OF reduced the TGFβ1-induced migratory ability and tube-forming capacity of endothelial cell lines in-vitro, hence curcumin inhibited the TGFβ1 mediated pro-angiogenic effect on TAO-OF.

### Gypenosides

Gypenosides are saponins and represent the most pharmacologically active component of *Gynostemma pentaphyllum*. Their biological actions include: regulating the activation of immune cells and the expression of cytokines [75]; decreasing inflammatory response in inflammatory bowel disease by inhibiting NF-κB and signal transducer and activator of transcription 3 (STAT3) signal pathways [76]; and inhibiting differentiation of hepatic progenitor cells into myofibroblasts and hence hepatic fibrosis by inhibiting the expression of TGF-β1, TGF-β1 receptor 1 and SMAD2/3 [77, 78].

Gypenosides displayed anti-inflammatory, anti-fibrotic and anti-oxidative effects in two separate in-vitro studies employing TAO-OF [75, 79]. Pretreatment with gypenosides significantly attenuated IL-1β-induced expression of pro-inflammatory cytokines (IL-6, IL-8, TNFα, CCL2) by TAO-OF, via reducing activation of Toll-like receptors 4 (TLR4)/NF-κB signaling pathway. TGFβ1 induced upregulation of fibrotic markers (hyaluronic acid, α-SMA, collagen type 1, fibronectin) in TAO-OF was prevented by gypenosides, through inhibiting SMAD2/4 signaling pathway [75].

In another study [79], gypenosides decreased oxidative stress via NRF2/ERK/HO-1 signaling pathway, as well as inhibited autophagy and apoptosis in TAO-OF treated with H2O2. They enhanced H2O2-stimulated malondialdehyde production but attenuated H2O2-induced SOD expression, suggesting regulation in the level of oxidative stress in TAO-OF. Gypenosides further promoted H2O2-induced expression NRF2/ERK/HO-1 proteins, while they inhibited H2O2-induced autophagy, as evidenced by reduced expression of autophagy activation-related proteins and reduced number of autophagosomes/autophagolysosomes. The anti-apoptotic effect was suggested by reducing the expression of apoptosis-related mRNA (caspase 3, BAX) and apoptosis rate of TAO-OF treated with H2O2. In addition, through in silico methods (including gene ontology analysis, protein–protein interaction network construction, and molecular docking), gypenosides might play an anti-inflammatory and anti-oxidative role in TAO via STAT1/3 signaling pathways [80]. Nonetheless, the therapeutic potential of gypenosides needs to be verified by further in-vitro and in-vivo studies.

### Dihydroartemisinin

Dihydroartemisinin is a derivative of artemisinin, a sesquiterpene lactone extracted from the Chinese herb *Artemisia annua L*. It is widely used in the treatment of fever and malaria [81]. Many in vivo experiments in disease-relevant animal models demonstrated the therapeutic efficacy of artemisinin-type drugs against rheumatic diseases, inflammatory bowel disease, and other inflammatory and autoimmune diseases [82, 83]. Apart from their antimalarial efficacy, artemisinin compounds demonstrated remarkable antifibrotic properties in multiple preclinical disease models [81]. For instance, dihydroartemisinin inhibited pulmonary inflammation/fibrosis and arthritis in animal models by suppressing JAK2/STAT3 signaling pathway [84, 85]. In an SLE mouse model, dihydroartemisinin ameliorated disease manifestation by inhibiting the senescence of myeloid-derived suppressor cells via activating NRF2/HO-1 signaling pathway [86]. Furthermore, dihydroartemisinin was shown to attenuate lipopolysaccharide-induced acute kidney injury via the inhibition of inflammatory mediators and oxidative stress [87].

Dihydroartemisinin demonstrated potent anti-inflammatory and antifibrotic effects on TAO-OF [88]. It reduced HA production and mRNA expression of pro-inflammatory cytokines, chemokines (IL-6, IL-8, CXCL-1, MCP-1, ICAM-1), as well as HA synthases, suggesting inhibition of IL-1β induced inflammation. Dihydroartemisinin inhibited proliferation, migration capacity and wound-healing ability of TAO-OF. Dihydroartemisinin significantly downregulated TGFβ1-induced expression of fibrosis markers (e.g. α-SMA, CTGF) at both mRNA and protein levels. As dihydroartemisinin decreased TGFβ1-induced phosphorylation of ERK1/2 and STAT3, its anti-fibrotic action was likely mediated via suppression of ERK and STAT3 signaling pathways.

### Berberine

Berberine, an isoquinoline alkaloid extracted from the Chinese herb *Coptidis rhizoma* (Huanglian), demonstrated multiple biological functions, including anti-inflammatory and anti-bacterial effects, alleviation of liver fibrosis, inhibition of carcinogenesis, cardiometabolic protection, as well as neuroprotection [89–92].

Berberine exerted inhibition on TAO-OF in an in-vitro experiment by suppressing inflammation, HA production, fibrosis, adipogenesis [93]. It attenuated IL-1β-induced expression of pro-inflammatory mediators (IL-6, PTX-3, and COX-2) via inactivating NF-κB signaling pathway. Berberine also inhibited TGFβ1 induced HA production and mRNA expression of HA synthases. Various fibrotic markers were downregulated at both mRNA and protein levels. A reduction of intracellular fat accumulation and various adipogenic markers at both mRNA and protein level was evident when TAO-OF undergoing adipogenic differentiation were treated with berberine. The suppression of adipogenesis was mediated through inhibiting AMPK and PPARγ signaling pathways.

Hydroxypropyl-berberrubine, an analog of berberine metabolite, significantly decreased cholesterol level by upregulating low-density lipoprotein receptor (LDL-R) and downregulating proprotein convertase subtilisin/kexin type 9 (PCSK9) in-vitro. Hypercholesterolemia itself was associated with increased risk and disease activity, as well as decreased response to intravenous glucocorticoid in TAO [94–96]. Statin, a class of well-established cholesterol-lowering agents, inhibited TAO-OF in-vitro [97–99], and atorvastatin was recently shown in a randomized controlled trial to enhance the clinical efficacy of intravenous glucocorticoid therapy in patients with active moderate-to-severe TAO [100]. Therefore, berberine may be beneficial in the management of TAO. Furthermore, the efficacy of berberine in Graves’ hyperthyroidism was also evaluated in a small non-randomized clinical trial [101]. Compared with methimazole monotherapy, the addition of berberine was associated with higher chance of normalizing TSH level and lower TSH-R-Ab levels at 6 months. Combination treatment also significantly modified the composition of gut microbiota, with an increased abundance of beneficial bacteria *Lactococcus lactis* and a reduced abundance of pathogenic bacteria *Enterobacter hormaechei* and *Chryseobacterium indologenes.* The potential beneficial effects of berberine on Graves’ hyperthyroidism and TAO will need to be further evaluated in larger clinical trials.

## Potential roles of TCM in Graves’ hyperthyroidism

Given the potential therapeutic effects of various TCM compounds on TAO and the shared pathogenesis between GD and TAO, TCM compounds could also be beneficial in the clinical management of Graves’ hyperthyroidism. Compared with antithyroid drug (ATD) monotherapy, ATD/TCM herbal combination more effectively reduced goiter size and relieved hypermetabolic symptoms in small clinical trials. Combined treatment may also offer both control of hyperthyroidism and desensitization in the setting of ATD allergy [102]. Acupuncture, an important TCM technique, is probably the most popular alternative therapy practiced in western countries, and its anti-inflammatory and immunomodulatory properties may also have potential therapeutic roles in TAO [103].

## Conclusions

Various active ingredients in TCM demonstrated inhibition of TAO-OF in-vitro and improved TAO in mouse models in-vivo. The growing interaction between conventional western medicine and TCM will help identify the effective molecules in herbal medicine with therapeutic implications and define precisely their role in clinical management [103]. Clinical trials of different phases with adequate power and sound methodology will be warranted to evaluate the appropriate dosage, safety and efficacy of these compounds in the management of TAO.
